# Synthesis, characterization, and cytotoxicity of doxorubicin-loaded polycaprolactone nanocapsules as controlled anti-hepatocellular carcinoma drug release system

**DOI:** 10.1186/s13065-022-00888-w

**Published:** 2022-11-12

**Authors:** Abdelgawad Fahmi, Mariam Abdur-Rahman, Omnia Mahareek, Mohamed A. shemis

**Affiliations:** 1 Chemistry Department, Faculty of Science, Cairo University, Giza, 12613 Egypt; 2 Biochemistry and Molecular Biology Department, Theodor Bilharz Research Institute, Giza, 12411 Egypt

**Keywords:** Doxorubicin, Polycaprolactone, Hepatocellular carcinoma, Cardiotoxicity, Nanocapsules

## Abstract

**Background:**

Free doxorubicin (Dox) is used as a chemotherapeutic agent against hepatocellular carcinoma (HCC), but it results in cardiotoxicty as a major side effect. Hence, a controlled Dox drug delivery system is extremely demanded.

**Methods:**

Dox was loaded into the non-toxic biodegradable polycaprolactone (PCL) nanocapsules using the double emulsion method. Characterization of Dox-PCL nanocapsules was done using transmission electron microscopy and dynamic light scattering. Encapsulation efficiency and drug loading capacity were quantified using UV–visible spectrophotometry. Drug release was investigated in vitro at both normal (7.4) and cancer (4.8) pHs. Cytotoxicity of Dox-PCL nanocapsules against free Dox was evaluated using the MTT test on normal (Vero) and hepatic cancer (HepG2) cell lines.

**Results:**

Spherical nanocapsules (212 ± 2 nm) were succeffully prepared with a zeta potential of (-22.3 ± 2 mv) and a polydisperse index of (0.019 ± 0.01) with a narrow size distribution pattern. The encapsulation efficiency was (73.15 ± 4%) with a drug loading capacity of (16.88 ± 2%). Importantlly, Dox-release from nanocapsules was faster at cancer pH (98%) than at physiological pH (26%). Moreover, although Dox-PCL nanocapsules were less toxic on the normal cell line (GI 50 = 17.99 ± 8.62 µg/ml) than free Dox (GI 50 = 16.53 ± 1.06 µg/ml), the encapsulated Dox showed higher toxic effect on cancer HepG2 cells compared to that caused by the free drug (GI 50 = 2.46 ± 0.49 and 4.22 ± 0.04 µg/ml, respectively).

**Conclusion:**

The constructed Dox-PCL nanocapsules constitute a potentially controlled anti-HCC therapy with minimal systemic exposure.

**Graphical Abstract:**

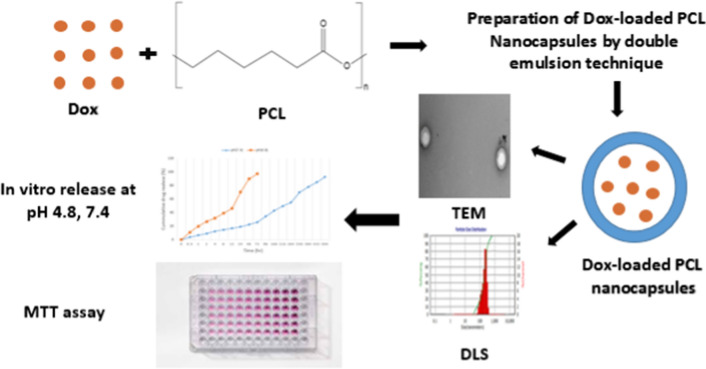

## Background

Globally, liver cancer is considered as the second cause of death with an annual incidence of ~ 850,000 cases [1]. In Egypt, liver cancer is considered as the most common type of cancer in males and the second most common type of cancer in females [2, 3].

Hepatocellular carcinoma (HCC) represents 90% of all primary liver cases. Worldwide, HCC represents the seventh most common cancer in females and the fifth in males [4]. In Egypt, liver cancer constitutes 1.68% of total cancer cases and HCC accounts for 70.48% of all liver tumors [5].

Cirrhosis is caused by many factors such as infection with hepatitis B or hepatitis C viruses, alcoholism, inherited metabolic diseases, diabetes, smoking as well as exposure to aflatoxins: a group of mycotoxins produced by the *Aspergillus* fungus in foodstuffs like corn and peanuts during storage in warm damp conditions [6].

HCC treatment requires careful screening and selection of therapies such as radiotherapy, chemotherapy, and surgery. Although radio- and chemotherapeutic drugs are capable of shrinking tumor growth for a short time, they require multiple schedules of treatment. In addition, chemotherapeutic drugs do not only kill cancerous cells but also the healthy normal ones, resulting in significant toxic side effects for the patients [7].

Examples of these cytotoxic chemotherapeutic drugs are doxorubicin (Dox), mitomycin C, cisplatin, and mitoxantrone [8]. Dox (C_27_H_30_ClNO_11_) is an anthracycline antibiotic, which is widely used for the treatment of many types of tumors such as acute leukaemia, liver, lung, breast, ovarian, stomach, uterine, testicular and bladder cancers [9].

Several mechanisms have been proposed and subjected to controversy. One of the most popular explanations is the ability of Dox to inhibit DNA synthesis through intercalating with the DNA topoisomerase II enzyme [10–12]. Dox inhibition of these enzymes causes failure of resolving the knotting and supercoiling of the DNA double strands during transcription, recombination, and cellular replication, which results in the death of the cancer cell or any other cell by inducing cell apoptosis [13].

However, many adverse events are caused by the administration of Dox including; anaemia, vomiting, skin pigmentation, diarrhea, dehydration, gastrointestinal tract bleeding, hyperuricemia and cardiotoxicity [14]. Cardiotoxicity is considered as the main obstacle of treatment using Dox [15]. The first case of cardiotoxicity caused by the repeated treatment of doxorubicin was presented as heart failure in 1967 [16]. The cardiotoxicity induced by Dox occurs through the accumulation of both cellular reactive oxygen species (ROS) and mitochondrial irons, as a result of the binding of Dox molecules to the cell membranes [17].

Mitochondria are then the most affected intracellular organelles following cellular exposure to Dox, which is accumulated in the mitochondrial membrane mainly due to its high afinity to bind cardiolipin, a mitochondrial membrane phospholipid [18–21]. Cardiolipin is responsible for maintaining the structure, function, cardiac energy metabolism of the mitochondria and cell survival [22, 23]. Binding of cardiolipin to Dox results in the bloking of the mitochondrial electron transport chain through the deactivation of complexes I, III, and IV [24, 25]. Moreover, superoxide anion (O_2_^−^) is produced upon the reoxidation of these species via transferring one electron to molecular oxygen (O_2_). Furthermore, hydrogen peroxide (H_2_O_2_) is synthesized by manganese superoxide dismutase, and can undergo subsequent Fe or Cu mediated conversions into more reactive hydroxyl radicals [26, 27]. Consequently, these reactive species can cause lipid peroxidation as well as nucleic acid and protein oxidation. Hence, serious destructive effects in mitochnodria are resulted including damage of mitochondrial DNA (mtDNA), decrease of ATP concentrations, cardiolipin peroxidation, and alteration in the permeability of the inner mitochnondrial membrane [28–30].

In addition, tumor cells exhibited multidrug resistance (MDR), attributed to the presence of P-glycoprotein (P-gp), which can pump Dox out, resulting in reducing its intracellular accumulation and therapeutic efficacy. These drawbacks of Dox efficiency on cancer therapy resulted in the restriction of its clinical applications and the need to develop new drug formulations [18].

The advances in nanotechnology and nanomedicine have led to the use of nanocarriers as drug delivery systems to deliver a certain chemotherapeutic drug to cancer cells [19]. This is actually the main reason why much effort has been dedicated to the development of pharmaceutical and colloidal forms encapsulating this drug, to reduce its undesirable side effects [22].

The ideal drug delivery system (DDS) should have the ability to deliver the appropriate therapeutic drug dose to the target cells and release it in a controlled manner in the human body in order to maintain its applicable concentration for a certain period of time. This helps to diminish the side effects of drugs and maximize their therapeutic efficacy [24].

Among a range of drug nanoparticles that are synthesized using polymers are Polymeric Nanocapsules, which have a central core surrounded by a polymeric outer shell. These are mostly applicable as nanocarriers to deliver anticancer drugs. Moreover, they are able to incorporate higher doses of chemotherapeutic drugs, which results in the enhancement of their therapeutic effects [26, 28].

Recently, many scientists prepared different forms of Dox-loaded polymeric nanocapsules, using various polymers to minimize its toxic side effects [29].

In this study, polycaprolactone (PCL) was chosen to produce polymeric nanocapsules, as it is considered as an interesting biocompatible and biodegradable synthetic polymer for the preparation of nanocarriers with potential therapeutic applications. PCL is approved by the United States Food and Drug Administration (FDA) and commonly used to encapsulate a wide range of anticancer drugs [32].

The aim of the present study was the synthesis of polymeric nanocapsules with an aqueous inner core enclosed by an organic layer and both are surrounded by an outer aqueous shell. This double-layer structure can readily guarantee the incorporation of the anticancer Dox drug into the nanocapsules cores, whereas the hydrophilic outer shell can provide stabilization for the nanocapsules without the need for additional surfactant. The prepared Dox polymeric nanocapsules are to be highly promising for safe chemotherapeutic applications by reducing its cytotoxicity against normal Vero cell line, and increasing its therapeutic effect against cancer HepG2 cell line.

## Methods

### Materials

Polycaprolactone (Mw. 10 000 Da), polyvinyl alcohol (PVA) (Mw: 13,000–23,000 Da), potassium phosphate dibasic, and ethyl acetate were obtained from (Aldrich, UK).

Doxorubicin.HCl solution was purchased from (Ebewe Pharma, Australia). Dichloromethane (DCM) was from (Carlo, UK), Polyethylene glycol (PEG) (Mw: 8000 Da) was from (Fisher, USA), sodium chloride was from (Fluka, Germany), potassium chloride was from (Sigma, Germany), and sodium phosphate monobasic was from (Biobasic Company, Canada). The normal Vero cell line and liver cancer HepG2 cell line were purchased from the tissue culture laboratory of (VACSERA, Egypt). The 3-(4, 5-dimethylthiazol-2-yl)-2 and 5-diphenyltetrazolium bromide (MTT) was from (Sigma, USA), phosphate buffer saline (PBS) was from (Biowest Nuaille, France), DMEM Earle’s medium with L-Glutamine, HEPES and Pen-Strep antibiotics were purchased from (Lonza, Switzerland). Dimethylsulfoxide (DMSO) was purchased from (Sigma, Germany).

### Preparation of Dox—PCL nanocapsules

Dox-PCL nanocapsules were produced by modified double emulsion technique (W/O/W) as described by [33]. Briefly, 1.5 ml Dox solution (2 mg/ml) were emulsified with 10 mg PCL dissolved in 8 ml DCM using a high speed homogenizer (Tekmar, UK) for 3 min at 5000 rpm to create the first emulsion phase of water-in-oil (W1/O). Then, the first emulsion is transferred to an aqueous solution containing 35 ml 2% PVA and 5 ml 0.5% PEG and are homogenized for 5 min at 8000 rpm to form the second emulsion phase (W1/O/W2). The resulting mixture was left stirred on a magnetic stirrer (C-MAG HS 7, IKA, China) overnight at room temperature, in the dark. After evaporation of DCM, the remaining solution was centrifuged using an ultracentrifuge (supra25K, Hanil science industrial, Korea) for 1 h at 13,000 rpm and 10 ^◦^C. Finally, the supernatant was decanted and stored to be used later on (“Evaluation of Dox encapsulation efficiency and drug loading” section) and the pellet was resuspended in 2 ml deionized H_2_O and directly fed into the freeze dryer (Edwards Modulyo, UK) to produce a dried powder of Dox- PCL nanocapsules, which was then collected and kept at 4 °C.

### Evaluation of Dox encapsulation efficiency and drug loading

The encapsulation efficiency percentage (EE %) and drug loading content (DL%) of Dox-PCL nanocapsules were indirectly measured using the supernatant resulted from the previous step (“Preparation of Dox—PCL nanocapsules” section), i.e., after centrifugation of the final nanoemulsion mixture solution produced from the final phase of the double emulsion procedure used to prepare Dox-PCL nanocapsules at 13,000 rpm for 1 h. The sample was measured in triplicate using UV–Vis spectrophotometer (Model: se6100 UV–Vis double beam, Abbota Corporation, USA) at 480 nm and the results were presented as the mean ± SD. Standard calibration curve of known Dox.HCl concentrations (1, 0.5, 0.25, 0.125, 0.0625, 0.03125, 0.0156, 0.0078, 0.0039, 0.00195, 0.000975 and 0.0000487) mg/ml was constructed [34]. EE % and DL % were calculated using the following Eq. (A.1) and Eq. (A.2):

A.1 $$EE({\%})=1-\frac{conc\,of\,free\,Dox}{conc\,of\,total\,Dox} \times 100$$

A.2 $$\text{DL}\,({\%})=\frac{\text{M}\;\text{of}\;\text{Dox}\times \text{EE}}{\text{M}\;\text{of}\;\text{Dox}+\text{M}\;\text{of}\;\text{Polymer}} \times 100$$

where, M of Dox and M of polymers are the initial mass of Dox and PCL, respectively, used in the double emulsion technique.

### In vitro release study of Dox from PCL nanocapsules

The released amount of Dox from the polymeric nanocapsules was investigated at two pH values in simulated dissolution media: pH 4.8 simulating the pH of cancerous cells, and pH 7.4 simulating the environment of the normal tissues, over a period of 23 days according to [35, 36]. Briefly, 1-mg sample of Dox-PCL nanocapsules was added to a 5-ml volume of 1X PBS at (pH 7.4) or (pH 4.8) and incubated in an orbital shaker stirring at 37 °C and 100 rpm. A 500-μL aliquot of the supernatant was taken at different time intervals (pH 4.8: 0.5, 1, 2, 4, 6, 12, 24, 48 and 72 h; pH 7.4: 0.5, 1, 2, 4, 6, 12, 24, 48, 72, 96, 168, 216, 264, 336, 384, 432, 504 and 552 h) and supplemented with a 500-μL fresh 1X PBS solution to maintain the total volume. The absorbance of released Dox was measured at 480 nm using UV–Vis spectrophotometer. Two standard calibration curves of known Dox.HCl concentrations (1, 0.5, 0.25, 0.125, 0.0625, 0.03125, 0.0156, 0.0078, 0.0039, 0.00195, 0.000975 and 0.0000487) at the two pH values (4.8 and 7.4), were separately constructed. Finally, the cumulative release of Dox from PCL nanocapsules was calculated using Eq. (B.1).

B.1 $$\text{The}\;\text{cumulative}\;\text{drug}\;\text{release}({\%})=\frac{\text{amount}\;\text{of}\;\text{Dox}\;\text{released}}{\text{initial}\;\text{amount}\;\text{of}\;\text{Dox}}\times 100$$

### Morphology of Dox-PCL nanocapsules

The shape of the nanocapsules was determined using a transmission electron microscope (TEM) (JEM-1400, Jeol, USA). A small drop of the nanocapsules suspension was added to the carbon-coated grid, stained with uranyl acetate, and air-dried before measurement.

### Assessment of nanocapsule size and size distribution

The average size and PDI of the nanocapsules were measured using DLS (Malvern, UK). The sample was diluted and sonicated for 5 min and then measured at room temperature.

### Zeta potential analysis of the nanocapsules

The surface charges of the obtained nanocapsules were measured as a function of zeta potential by DLS (Nanotrac, wave2, UK).

### In vitro studies of Dox-PCL nanocapsules on the viability of normal and cancer cell lines

Normal Vero cell line and liver cancer HepG2 cell line were chosen to evaluate the cytotoxicity of both free Dox and Dox-PCL nanocapsules by MTT assay. The cells were cultured according to the protocol proposed by American Type Culture Collection (ATCC) [37]. HepG2 and Vero cells were cultured separately in 6-well cell culture plates at a density of 30000 cells per well for 24 h at 37 °C in a 5% CO_2_ incubator. Based on the study [38], various concentration ranges of the free drug, Dox, have been prepared (42.2 to 0 µg/ml). Equivalent amounts of encapsulated drug inside drug-containing PCL nanocapsules (250 to 0 µg/ml) were adjusted to the same concentrations of the free drug by the corresponding cell culture medium. The MTT assay was done based on the calculation of the drug loading capacity of anticancer drug, Dox, inside PCL nanocapsules: Each 1 mg of the nanocapsules contains 0.1688 mg/ml of Dox. For example, the first concentration of Dox-PCL nanocapsules 250 μg/ml contains 42.2 μg/ml of the free Dox as shown in Table 1*.*

**Table 1:** The concentrations of Dox and Dox-PCL nanocapsules used in MTT assay

Dox volume (µl) used as a positive control	Dox conc. (µg/ml)	Equivalent Dox conc. inside Dox-PCL nanocapsules (µg/ml)
21	42.2	250
10.5	21.1	125
5.26	11	62.5
2.63	5.3	31.25
1.315	2.6	15.6
0.657	1.3	7.8
0.329	0.65	3.9
0.164	0.325	2
0	0	0

Briefly, cells were then treated for 72 h with Dox-PCL nanocapsules solution at concentrations of (250, 125, 62.5, 31.25, 15.6, 7.8, 3.9, 2, 1 and 0) µg/ml. Free Dox drug was used as a positive control at concentrations of (42.2, 21.1, 11, 5.3, 2.6, 1.3, 0.65, 0.325, 0) µg/ml. Then, 20 µl of MTT reagent (5 mg/ml) was added to each well and the plates were incubated for 3 h at 37 °C in a 5% CO_2_ incubator. Finally, the media with MTT solution was carefully aspirated and replaced with 150 µl DMSO by a multichannel micropipette, the plates were covered with aluminum foil, and agitated on an orbital shaker for 15 min. Absorbance at λ 570 nm was measured using a microplate reader. The cell viability is expressed as a percentage of viability using Eq. (C.1):

C.1 $$\text{Viable}\;\text{cells}\;({\%})= \frac{{A}_{Treated}-{A}_{ Blank}}{{A}_{Control}-{A}_{ Blank}}$$

where, A_Treated_ is the average absorbance in wells containing cells treated with a defined concentration of free Dox or Dox-PCL nanocapsules, A_Blank_ is the absorbance of blank (DMSO), and A_control_ is the absorbance of the untreated cells that were used as negative control [39, 40].

In parallel, the cytotoxic effect of empty PCL nanocapsules (0 to 250 µg/ml) was also studied as a blank control.

For inhibitory concentration (GI 50) calculation of free Dox and Dox-loaded PCL, an excel sheet was used after calculating the inhibition percentage at various concentrations of both Dox forms and the results are expressed as mean ± SD.

### Statistical analysis

The data were presented as the mean ± SD of at least three replicates. The test of significance was performed by GraphPad Prism 7 (San Diego, California, USA). Two-tailed multiple T-test was employed to determine the significance of differences between normal and cancer cells. The ρ-value < 0.05 was considered to indicate a statistically significant difference.e.

## Results

### Calculation of EE % and DL % of Dox-PCL nanocapsules

**Fig. 1:**
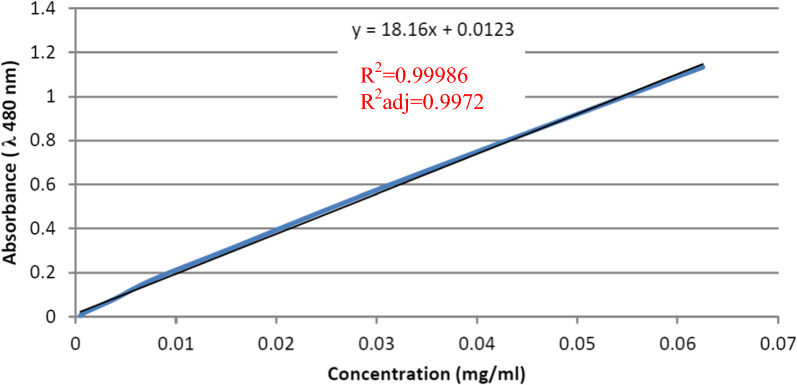
Calibration curve of known concentrations of Dox vs absorbance

Firstly, a calibration curve of known drug concentrations vs absorbance at λ 480 nm was constructed as shown in Fig. 1.

Dox-PCL nanocapsules were successfully prepared by the double emulsion technique. Encapsulation efficiency (EE %) and drug loading (DL %) were 73.15% ± 4 and 16.88 ± 2%, respectively.

### Characterization of Dox-PCL nanocapsules

**Fig. 2:**
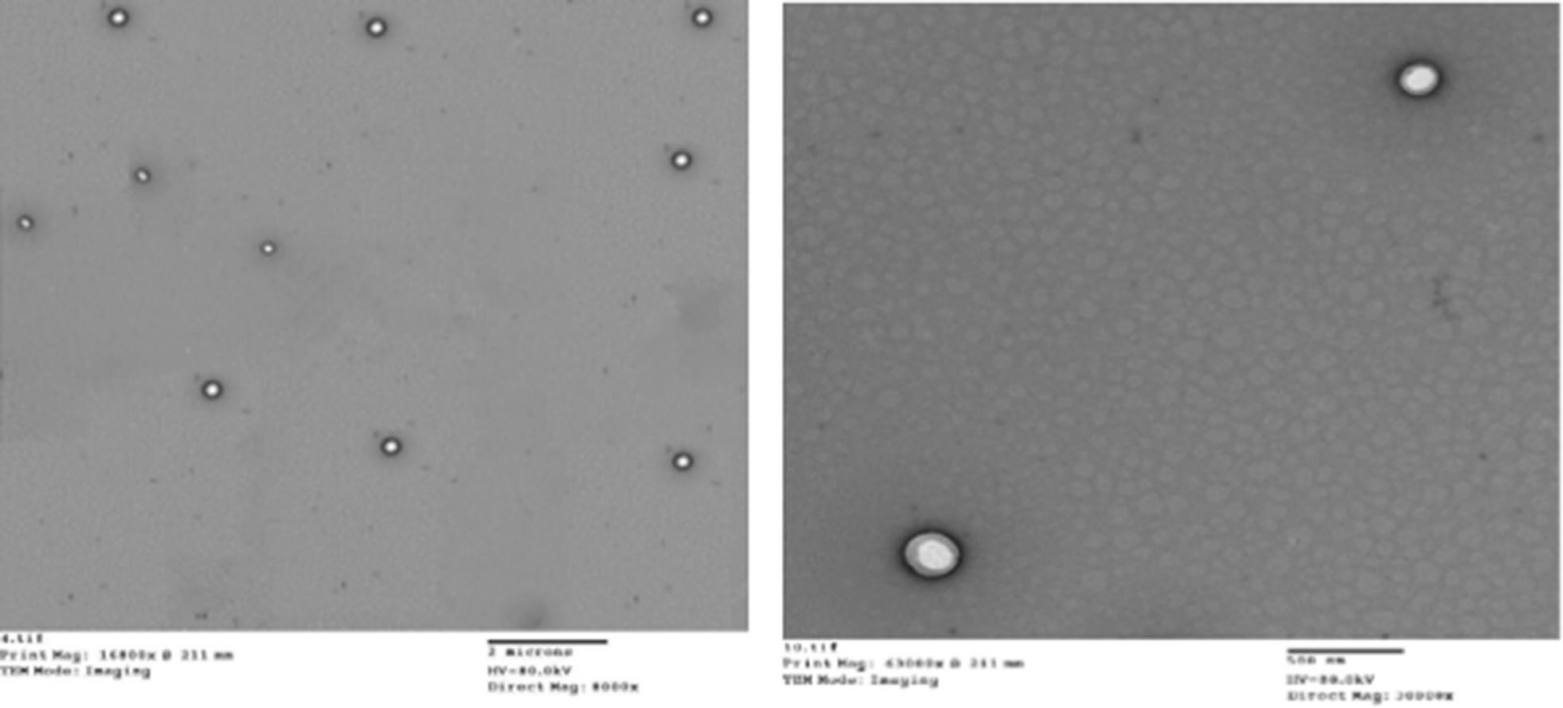
TEM image of Dox-PCL nanocapsules

**Fig. 3:**
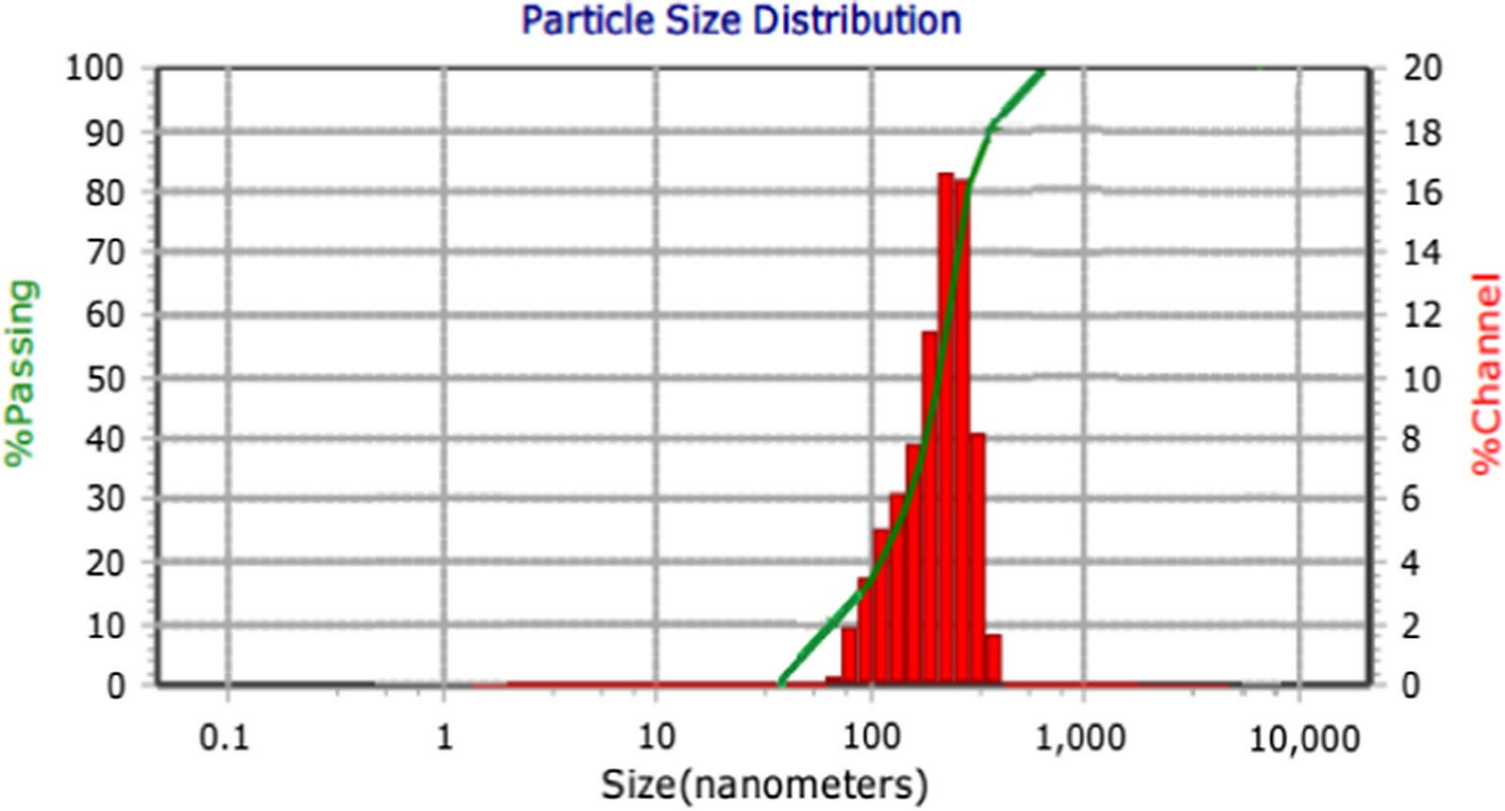
Particle size distribution of Dox-PCL nanocapsules

A TEM image revealed that Dox-PCL nanocapsules prepared by double emulsion were spherical as shown in Fig. 2. The nanocapsules appeared as bright spherical entities surrounded by a dark stain. It was apparent that Dox was assembled in the nanocapsule core surrounded by the hydrophobic PCL part and the aqueous phase of PVA was exposed to the outer shell. The particle size and PDI are illustrated in Fig. 3. The average size of nanocapsules determined by DLS was 212 nm ± 2, the zeta potential was -22.3 ± 2 mv, and PDI was 0.019 ± 0.01 with a narrow monodispersed unimodal size distribution pattern.

### Cumulative release of Dox from PCL nanocapsules at normal and cancer pHs

**Fig. 4:**
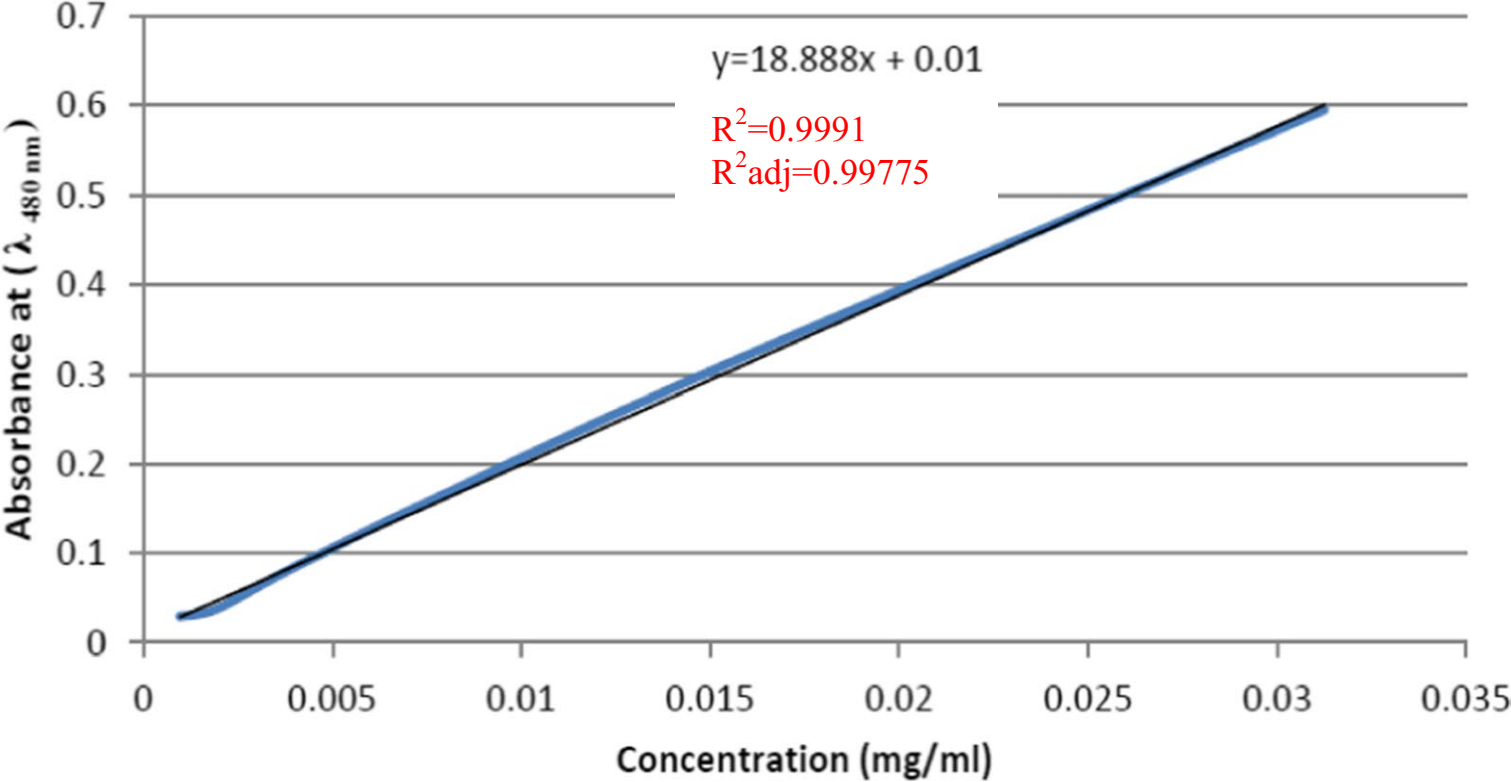
Calibration curve of Dox at pH 7.4

**Fig. 5:**
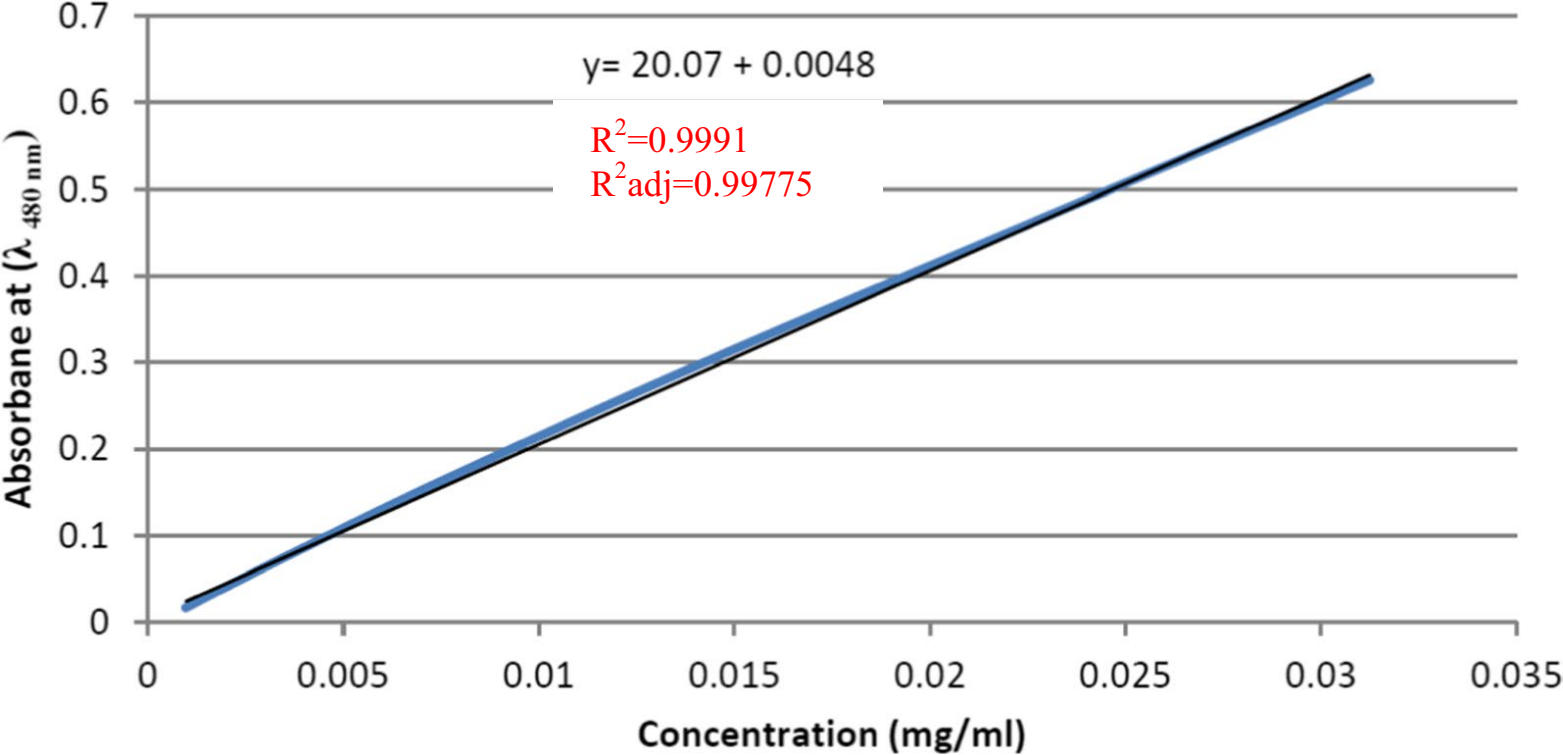
Calibration curve of Dox at pH 4.8

Two calibration curves of known drug concentrations vs absorbance at λ 480 nm were constructed at 2different pHs (normal 7.4; cancer 4.8) as shown in Figs. 4 and 5.

**Fig. 6:**
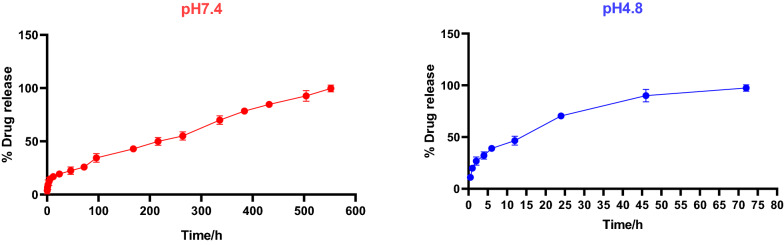
In vitro Dox-release profiles of PCL nanocapsules at pH 7.4 and pH 4.8; Data represent the mean ± SD, number of samples (n) = 3

The profile of Dox-release indicated only one phase of release as shown in Fig. 6. Approximately 97.45% of Dox was released within 72 h at pH 4.8 (cancer pH). Whereas, the rate of Dox release at pH 7.4 (normal pH) was approximately 99.67% over 23 days, which is notably slower than that at the cancer pH.

### In vitro cytotoxicity evaluation of free Dox, blank PCL and Dox-PCL nanocapsules by MTT assay

**Fig. 7:**
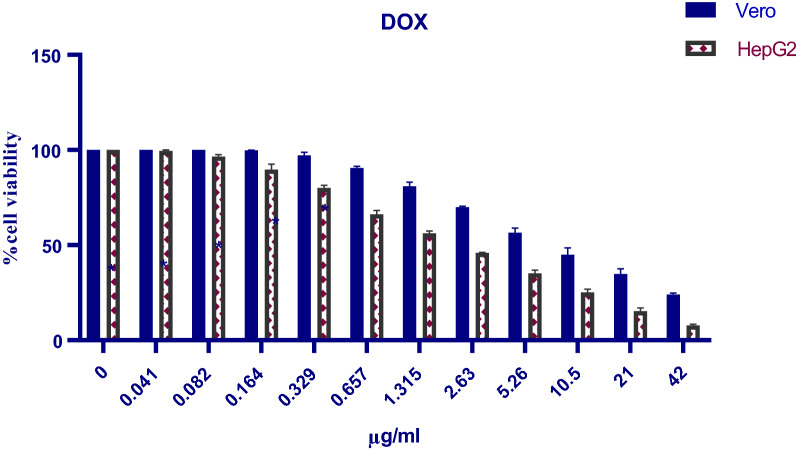
The cytotoxicity of free Dox on Vero and HepG2 cell lines

**Fig. 8:**
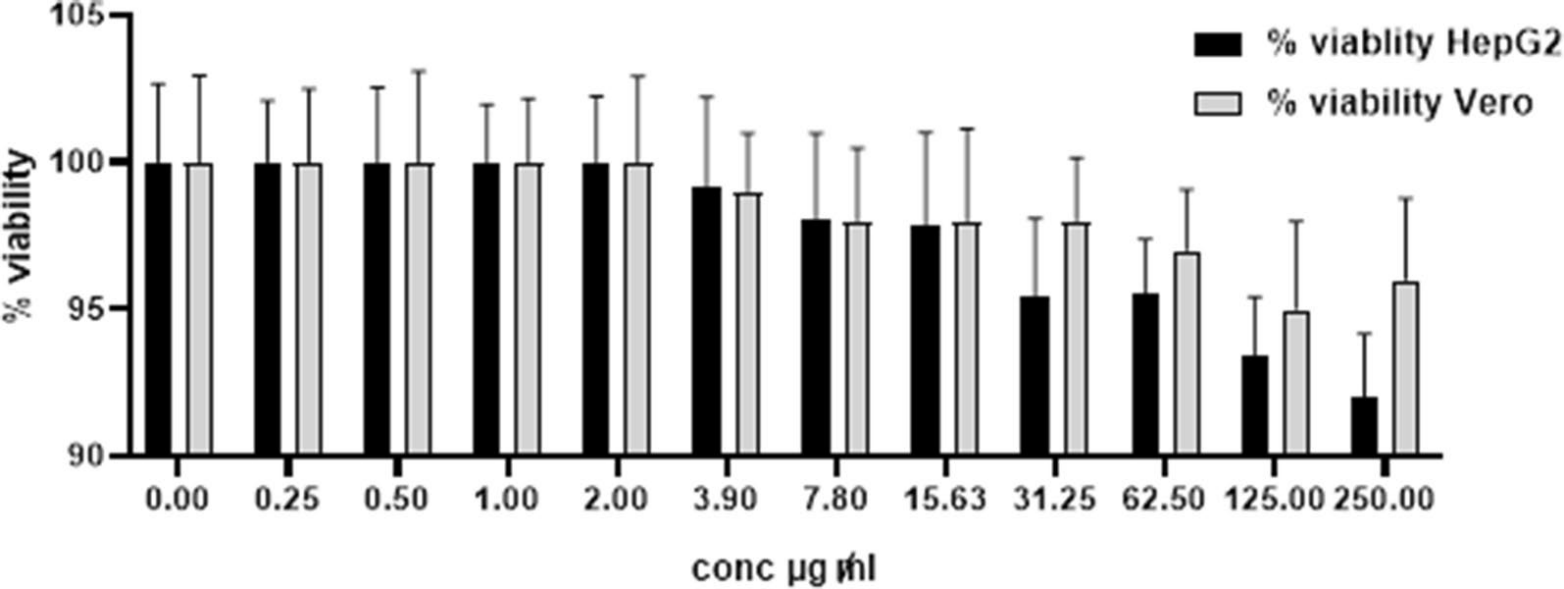
The cytotoxicity of blank PCL nanocapsules on Vero and HepG2 cell lines

**Fig. 9:**
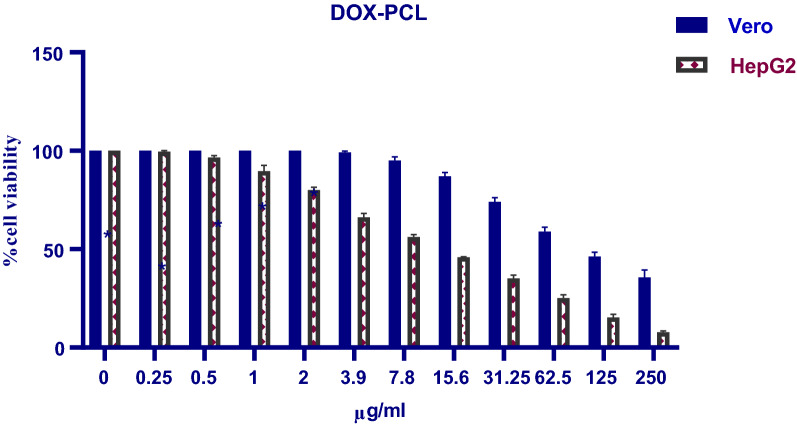
The cytotoxicity of Dox-PCL nanocapsules on Vero and HepG2 cell lines

Results shown in Fig. 7 illustrated that after 72 h treatment with free Dox (42.2–0 µg/ml), normal Vero cells showed a viability percentage of 34.77–100%, while cancer HepG2 cells had a viability percentage of 15.31–100%. Application of empty blank PCL nanocapsules (250–0 µg/ml) resulted in vialbility percentages of ~ 97–100% and ~ 93–100% in Vero and HepG2 cell lines respectively (Fig. 8). On the other hand, Dox-PCL nanocapsules (250–0 µg/ml) containing equivalent amounts of the drug (Table 1) caused normal Vero cells to show a viability percentage of 35.72–100% while cancer HepG2 cells showed a viability percentage of 11.06–100% (Fig. 9). Statistical analysis using Multiple T-test showed a high significance between normal and cancer cells at all tested concentrations of Dox, blank PCL and Dox-PCL nanocapsules (*p*-value < 0.001).

**Fig. 10:**
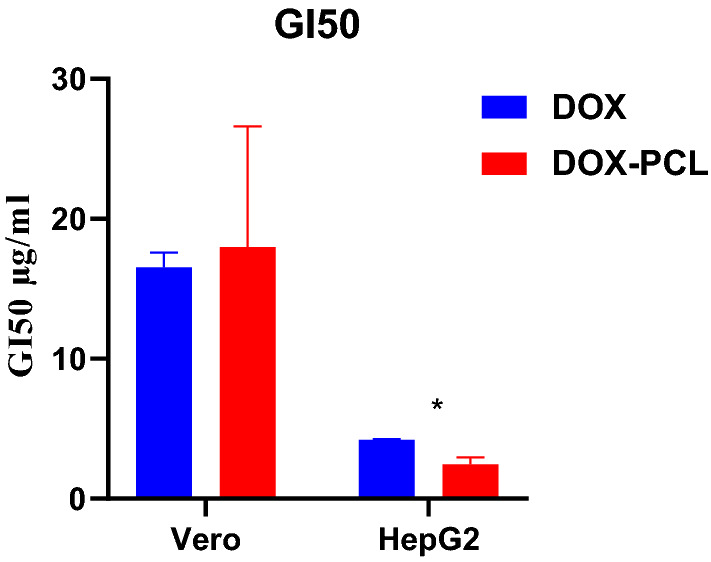
Comparison between the (GI 50) values of free Dox and Dox-PCL nanocapsules against Vero and HepG2 cell lines

The GI 50 (inhibitory concentration to cause growth inhibition by 50%) of free Dox and Dox-PCL nanocapsules was also studied as shown in Fig. 10. For normal Vero cells, the results indicated that GI 50 of Dox-PCL was 17.99 ± 8.62 µg/ml after 72 h as compared with free Dox that had GI 50 of 16.53 ± 1.06 µg/ml. On the other hand, the GI 50 of both 72 h treatments with free Dox and Dox-PCL on HepG2 cells were 4.22 ± 0.04 and 2.46 ± 0.49 µg/ml, respectively, Statistical analysis showed that there was a statistical significance between free Dox and Dox-PCL nanocapsules (*p*-value = 0.0034).

## Discussion

Dox is a chemotherapeutic drug extremely used in the treatment of many types of cancer. Its mechanism of action is based on binding to the DNA strands to inhibit its macromolecular synthesis. The disadvantages of Dox-based cancer therapy include the fact that Dox has toxic side effects on both cancer and healthy cells causing heart failure. In addition, tumor cells exhibited multidrug resistance, attributed to the presence of P-glycoprotein, which can pump Dox out, resulting in reducing its intracellular accumulation and decreasing its therapeutic efficacy. These are the major drawbacks of Dox efficiency against cancer therapy which results in restriction of its clinical applications and the need to develop new drug formulations [41]. These requirements have resulted in a trend towards miniaturization, which has challenged scientists from multidisciplinary fields to engineer novel drug delivery systems. Furthermore, several drug molecules cannot be formulated or administered by conventional techniques as they exhibit poor encapsulation efficiency of hydrophilic anticancer drugs such as Dox or suffer from limited sustained release in a complex environment such as the human body [42].

The ideal drug delivery system (DDS) should be capable of delivering a wide range of anticancer drugs for a sustained period of time into the target sites in a slow release manner. This provides an enhanced antitumor efficacy with reduced systemic side effects. Moreover, these DDS can protect drugs against their rapid metabolism and clearance by the liver, kidneys, and reticuloendothelial system, which then enhance drug stability and target specificity [22, 43, 44].

Due to their exceptional properties compared with bulk materials, nanoparticles applications are now found in various practical fields. In this regard, a combination of pharmacology and nanotechnology has contributed to produce more effective anticancer agents by decreasing the resistance of cancer cells. There are many types of metallic nanoparticles with several physicochemical properties such as selenium, silver, gold, titanium dioxide, zinc oxide, copper oxide, platinum, and magnesium oxide, which have been well known as anticancer carriers [45]. However, metallic drug nanocarriers result in raising issues of toxicity to humans and the environment. The insoluble metallic nanoparticles are likely to be accumulated in sensitive organs such as the heart, liver, spleen, kidney, and brain after inhalation, ingestion, and skin contact. Also, *in vitro* and *in vivo* studies had provided that the exposure to metallic nanoparticles could induce the production of reactive oxygen species (ROS), which is a predominant mechanism leading to organ toixicity [46]. Thus, efforts are focusing on using biocompatible proteins and polymers such as albumin nanoparticles [47]. But, the major obstacles associated with using albumin as DDS are; low stability, poor batch-to-batch reproducibility, difficulty in sterilization, only used with hydrophobic drugs, and low drug loading capacity. Therefore, albumin is not suitable to encapsulate the highly hydrophilic anticancer Dox.HCl drug [48]. As the selection of a suitable polymer is a crucial step, polycaprolactone (PCL) was chosen in our study to produce polymeric nanocapsules. PCL is a biodegradable and biocompatible synthetic aliphatic polyester approved by FDA. PCL and its derivatives are nontoxic and have high permeability to a lot of drugs. Properties of PCL like thermal responsiveness, drug entrapment, degradation kinetics, mechanistic properties, fabrication easiness, and controlled release nature make it a promising polymer for colloidal drug delivery applications. Most importantly, PCL is a very hydrophobic crystalline polymer, which is broken down by hydrolysis of its ester bonds under the normal physiological conditions in the human body and has minimal or no toxicity. Therefore, it has taken the attention of researchers as an applicant of choice for use in drug delivery applications and long-term implantable devices for both lipophilic and hydrophilic drugs [49]. In this study, the biocompatible and nontoxic PCL polymeric nanocapsules were successfully loaded with Dox by a modified double emulsion technique (W/O/W) according to Katata et al*.* [33]. The double emulsion technique was chosen to encapsulate the hydrophilic Dox.HCl inside the first (inner) aqueous phase of the core–shell nanocapsules to provide a high drug loading content (16.68 ± 2%) as well as a specific and sustained release of the anticancer drug at the cancer cells only. This leads to the reduction of its damaging side effects on normal cells, and then enhances its therapeutic applications [50]. According to [33], the hydrophilic Dox.HCl, a toxic chemotherapeutic drug model, was loaded into PCL nanocapsules using the double emulsion (W1/O/W2) method. The compatibility between Dox and PCL were approved, whereas the ability of the inner core to encapsulate Dox is largely dependent upon the compatibility between the hydrophobic polymer and the drug molecule [51]. Furthermore, our results are in line with [52, 53], who selected the double emulsion technique due to its advantageous capability to encapsulate both hydrophilic and hydrophobic drugs using PCL.

For intravenous administration, nanomedicines with high drug loading content of more than 10% are favorable for cancer therapy, to reduce the drawbacks associated with nanocarrier materials having low drug content [54]. The prepared Dox-PCL nanocapsules were analyzed using DLS and were found to have a size of 212 ± 2 nm, a charge (ZP) of -22.3 ± 2 mv, and PDI of 0.019 ± 0.01 with a narrow monodispersed unimodal size distribution pattern. TEM imaging revealed that the prepared nanocapsules were spherical as shown in Fig. 2. Previous studies have shown that the shape of nanoparticles plays an important role in the delivery of the anticancer drug, especially in biological practices, including internalization by passage through the blood circulation system and directing to the sites of cancer [55]. Transport of spherical nanoparticles is expected to be much easier because of their characteristic symmetry, whereas nonspherical nanoparticles may align or tumble in the presence of flow [56]. The size of nanoparticles used in drug delivery systems should be in the range of (10–250) nm, which is large enough to prevent their rapid leakage from blood capillaries but small enough to escape capture by fixed macrophages that are lodged in the reticuloendothelial system (RES), such as the spleen and liver [22, 57]. Moreover, zeta potential (ZP) is an important parameter to predict the storage stability of the nanoparticles colloidal suspension. High values of ZP, either positive or negative, are required to confirm stability and avoid aggregation of the particles by electrostatic repulsive forces [58, 59]. ZP of more than ± 13 mV indicates stable nanoparticles [60]. PDI is a numerical value that represents the homogeneity of the sample. If PDI is below 0.4, the particles of the sample are considered homogenous (i.e., similar size). If PDI is higher than 0.4, the sample is less homogenous. If PDI is more than 1, the sample is completely heterogeneous [61]. In this study, a pH of 4.8 was selected to mimic the pH of cancer cells, while a pH of 7.4 was selected to mimic the pH of the healthy cells. It was observed that the Dox release from the polymeric nanocapsules was faster (~ 97% within 72 h) at pH 4.8 than at pH 7.4 (~ 97% over 23 days), which is an excellent indicator of the selectivity of Dox-PCL nanocapsules to be released at the cancer site (pH 4.8), while Dox was hardly released at the normal site (pH 7.4). According to [62], a free Dox solution was used as a positive control, and showed a relatively fast release of Dox, reaching 100% release of the total theoretical amount of Dox at 2 h and 35 min at pH 7.4. Also in the study of [63], compared with the free Dox that released to the extent of 92% within 2 h, the Dox-loaded PLA–PEG–FA SPIONs showed sustained release at a steady rate of Dox by diffusing through the polymeric matrix for 120 h. In our study, the pH 4.8 was selected to mimic pH of cancer cells, while pH 7.4 was selected to mimic pH of the healthy cells. It could be observed that Dox release from Dox-PCL was faster at pH 4.8 than at pH 7.4. This was obvious also when the color of incubation media was changed after 12 h of incubation. During the releasing process, Dox was first released inside the hydrophobic core region of the polymeric nanocapsules, then diffused out from the hydrophilic outer shell of the nanocapsules, and eventually into the incubation medium. This delay of drug release indicates the nanoparticle applicability as a powerful drug carrier that minimizes the exposure of healthy tissues while increasing the accumulation of therapeutic drug in the tumor site resulting no more Dox-repeated treatment which was the main reason of cardiotoxicity.

Moreover, our results are significant to the study done by [64], which reported that in the in vitro release rate of Dox from Thermo/pH-responsive targeted polymeric nanocapsules (p(NIPA-co-AAc-co-GAA)), fabricated through double emulsion solvent evaporation technique with drug loading content of 24.31%, was much higher under acidic pH 3.0 than under physiological pH 7.4. Moreover, our results are advantageous over that from [65], in which magnetic nanoparticles were prepared based on cyclodextrin dendritic-graphene oxide as nanocarriers for Dox, with a drug loading capacity of only 9.8%, while our Dox-PCL nanocapsules showed a higher drug loading capacity reaching 16.88%. Moreover, our results are significant to [66], showing that the in vitro release rate of Dox from selenium nanoparticles (SeNPs) decorated with hyaluronic acid (HA), HA-SeNPs nanoparticles, was up to 76.9% at 30 h. Nevertheless, the release rate of Dox in PBS at pH 7.4 was about 53.5%. Our results are advantageous over that of [67], who prepared a magnetic iron oxide nanoparticles (IONPs) stabilized with trimethoxysilylpropyl-ethylenediamine triacetic acid (EDT) were developed as a nanocarrier for anticancer drug doxorubicin, with drug loading capacity of only 5 ± 0.05%, while our dox-PCL nanocapsules showed a higher drug loading capacity reaching 16.88%. Also, our results are advantageous over that from [68], in which Dox-PLGA-lecithin PEG biotin NPs were synthezised with drug loading content of only 7.21%, after transformation of hydrophilic Dox.HCl into hydrophobic Dox. The in vitro release studies showed that more than 50% of Dox were released from PLPB-NPs after 96 h of incubation at pH 7.4.

The effects of free Dox and Dox-loaded PCL nanocapsules on the viability of different cell lines, but not Vero and HepG2, were examined by the MTT assays, to evaluate their cytotoxicity and pharmaceutical efficacy. In our study, blank PCL nanocapsules have been studided in parallel and results showed that the empty vehicles were safe on normal Vero cells (97–100% viability) and had a negligible cytotoxic effect on HepG2 cells (93–100% viability). Which agrees with [69, 70], approving that the blank PCL nanocapsules have almost no cytotoxic effect on the normal Vero cell line. Also, according to [71], the PCL showed in vitro antiproliferative and antioxidant effects against HepG2 cancer cells while causing opposite action on healthy hepatocytes by increasing their survival until 4 weeks in culture. Hence, the effect of empty PCL nanocapsules could be neglected in our study for both the normal Vero and cancer HepG2 cell lines. Our results from MTT test reported that in comparison to free drug, Dox-PCL nanocapsules had a more therapeutic effect against the viability of HepG2 cancer cells but a less cytotoxic effect on normal Vero cells. In which, cancer cells were significantly more sensitive to encapsulated than free drug resulting in cell viability percentages of (11.06 to 100%) and (15.31 to 100%), respectively. In contrast, the viability of normal Vero cells was significantly increased after drug encapsulation (free Dox: 34.77 to 100%; Dox-PCL: 35.72 to 100%). These differences in cytotoxicity between free Dox and Dox-loaded nanocapsules could be attributed to the different cellular uptake mechanisms of the drug, in which the cellular uptake of free Dox occurs through a passive diffusion mechanism. This is reasonable since Dox could move freely through both the plasma membrane and nuclear membrane, which may result in the trapping of the drug at the P-gap junction counteractive effects in healthy cells. However, in the case of Dox-loaded nanocapsules, the drug is released in a time-dependent manner from the nanocapsules before applying its effect on the cells, thus drug release increases with time which results in increasing the concentration and inhibiting the cell growth [72, 73]. The mechanism of Dox release from the nanocapsules could be attributed to the acidic environment at the tumor cells resulting in the dissociation of Dox-loaded nanoparticles and rapid release of the Dox. As a small molecule, the uptake of free Dox is a dynamic process and it can freely escape from the cells, while the cellular uptake of the larger Dox-loaded nanocapsules is done through a non-specific endocytosis, which may lead to a reduced effect of cytosolic free Dox on the P-glycoprotein (P-gp) pumping action. Likely, the prolonged circulation and passive tumor targeting delivery process caused by the EPR effect could enhance the delivery of Dox into the tumor cells, and once the nanocapsules were internalized, it is not easy for them to escape from the cells. P-gp can recognize and eject the anticancer drug Dox from the tumor cells only when it is located in the plasma membrane, and not in the cytoplasm or lysosomes after endocytosis [74].

Our data also confirmed that after 72-h incubation, the GI 50 of Dox-PCL on normal Vero cells was higher than that of Dox used alone (Dox-PCL: 17.99 ± 8.62 µg/ml vs free Dox:16.53 ± 1.06 µg/ml). On the other hand, a higher cytotoxic effect (i.e., lower GI 50) against the hepatic cancer line was achieved in favour of the prepared nanocapsules compared to the free Dox (Dox-PCL: 2.46 ± 0.49 µg/ml vs free Dox: 4.22 ± 0.04 µg/ml).

## Conclusions

**Fig. 11:**
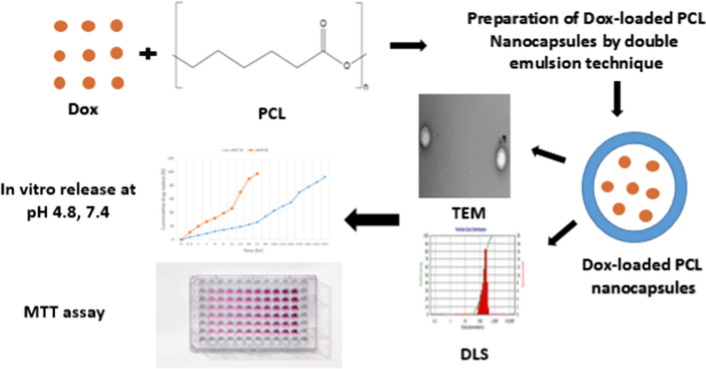
Graphical Abstract; Polycaprolactone (PCL)-based nanocapsules loaded with the antitumor agent doxorubicin (Dox) were prepared using the double emulsion technique. The promising nanocarriers (212 nm) were defined to have a narrow monodispersed unimodal size distribution with ZP of -22.3 mv, PDI of 0.019, and DL capacity of 16.88 %. The safety of the little cargo was ensured by having an enhanced drug release rate reaching 98 % at the cancer acidic medium (pH 4.8) compared to only 26 % at the physiological medium (pH 7.4), meaning less drug exposure to normal cells. Moreover, nanoencapsulation has effectively reduced Dox-cytotoxicity on normal Vero cells, while notably increasing the drug sensitivity of cancer HepG2 cells

Nontoxic polymeric Dox-loaded PCL nanocapsules were successfully developed by double emulsion technique (Fig. 11) with a high drug loading content of 16.18 ± 2%. A stable colloidal suspension of spherical Dox-PCL nanocapsules (particle size: 212 ± 2 nm; ZP: -22.3 ± 2 mv; PDI: 0.019 ± 0.01) with a narrow size distribution pattern was characterized. MTT test and GI 50 results confirmed that in comparison to equivalent amounts of free Dox, the prepared encapsulated drug exhibited a higher in vitro therapeutic cytotoxicity on cancer HepG2 cell line, while being less toxic on normal Vero cells. The Dox was released from Dox-PCL nanocapsules at pH 4.8 (cancer pH) faster than at pH 7.4 (healthy pH); 98%

and 26 over 72 h, respectively. Finally, the polymeric based drug delivery system offers a successful and promising potential application for many therapeutic agents with more confidence in Dox for the clinical treatment of hepatocellular carcinoma.

## Recommendations

Further in vivo studies as well as fabrication of Dox-PCL nanocapsules with both targeting (folic acid) and labeling (rhodamine) agents would be of useful importance in evaluating the potency and pharmacokinetics of these promising biocompatible Dox nanocarriers.

## Data Availability

The datasets used and/or analyzed during the current study are available from the corresponding author on reasonable request.

## Abbreviations

DCM: Dichloromethane
DDS: Drug delivery system
DE: Double emulsion
DL: Drug loading
DLS: Dynamic light scattering
Dox: Doxorubicin
Dox-PCL: Doxorubicin loaded polycaprolactone
EE: Encapsulation efficiency
FDA: United States food and drug administration
HCC: Hepatocellular carcinoma
GI 50: Inhibitory concentration to cause growth inhibition by 50%
MDR: Multidrug resistance
MTT: 3-(4, 5-Dimethylthiazol-2-yl)-2, 5- diphenyltetrazolium bromide test
RES: Reticuloendothelial system
ROS: Reactive oxygen species
PBS: Phosphate buffer saline
PCL: Polycaprolactone
PDI: Polydisperse index
PEG: Polyethylene glycol
P-gp: P-glycoprotein
PVA: Polyvinyl alcohol
TEM: Transmission electron microscopy
ZP: Zeta potential
